# Investigating mechanisms of cognitive control training: neural signatures of PASAT performance in depressed patients

**DOI:** 10.1007/s00702-021-02444-7

**Published:** 2021-11-23

**Authors:** Anja Sommer, Andreas J. Fallgatter, Christian Plewnia

**Affiliations:** Department of Psychiatry and Psychotherapy, Tübingen Center for Mental Health (TüCMH), Neurophysiology and Interventional Neuropsychiatry, University of Tübingen, Calwerstraße 14, 72076 Tübingen, Germany

**Keywords:** Major depressive disorder, Cognitive control, Cognitive control training, PASAT, dlPFC, Event-related potentials

## Abstract

Major depression disorder (MDD) is characterized by cognitive control (CC) dysfunctions associated with increased attention toward negative information. The paced auditory serial addition task (PASAT) has been used as a targeted training of CC and studies show promising effects on depressive symptoms. However, neural mechanisms underlying its efficacy are still unclear. Based on previous findings of feedback-locked event-related potentials in healthy subjects, we investigated neural signatures during PASAT performance in 46 depressed patients. We found significantly larger amplitudes after negative than positive feedback for the P300 and late positive potential (LPP). However, this difference was not significant for the feedback-related negativity (FRN). Moreover, no associations of valence-specific ERPs and PASAT performance nor depressive symptoms were found. This indicates that depressed patients seem unable to use neural activation in late feedback processing stages (P300, LPP) to adapt accordingly. Moreover, lack of valence-specific neural reaction in early feedback processing stages (FRN) might point toward emotional indifference in depressed patients.

Trial registration number: NCT03518749 Date of registration: May 8, 2018.

## Introduction

Flexible adaptation of cognitive resources according to internal goals is an important prerequisite of goal-directed human behavior. This top–down-driven cognitive control (CC) is impaired in depressed patients, resulting in difficulties disengaging from negative thoughts, emotions, and information (Baert et al. 2010). Moreover, CC deficits have been found to be associated with the development and maintenance of MDD (Gotlib and Joormann 2010; LeMoult and Gotlib 2019). Thus, trainings that directly target disrupted CC functions are promising new treatment methods. In cognitive control trainings (CCT), patients repeatedly perform tasks on various cognitive functions, such as working memory, processing speed, or continuous attention. Studies so far show promising results concerning the clinical utility of CCTs (for an overview, see Koster et al. 2017; Van den Bergh et al. 2018). A training task commonly used as CCT is the paced auditory serial addition task (PASAT; Gronwall, 1977). In the PASAT, digits have to be added serially, while difficulty adapts according to the performance of the patients (Siegle et al. 2007a, b). Working at the individual limit of cognitive functioning while continuously receiving performance feedback results in the engagement of working memory and processing speed capacities in a stressful and emotional task context. Indeed, studies typically find negative affect significantly increased after PASAT performance (Holdwick and Wingenfeld, 1999; Plewnia et al. 2015). Furthermore, the PASAT was found to activate the dlPFC (Lazeron et al. 2003) which is typically hypoactivated in depressed patients, especially when it comes to tasks that require top–down-driven CC of emotions (Siegle et al. 2007a, b; Fales et al. 2008). Efficacy of the PASAT for the reduction of depressive symptoms has already been demonstrated (Hoorelbeke et al. 2015; Lass et al. 2021; Siegle et al. 2007a, b; Siegle et al. 2014; for an overview, see Koster et al. 2017 and Van den Bergh et al. 2018). However, findings about the mechanisms underlying the effectiveness of the PASAT are still scarce. It is assumed that the activation of prefrontal brain areas in a stressful and emotion-inducing environment strengthens prefrontal control over emotion-related limbic areas (Siegle et al. 2007a, b). This assumption is bolstered by findings that the effectiveness of the PASAT increases with the enhancement of stress induction (Lass et al. 2021). Moreover, an increase of dlPFC activity by transcranial direct current stimulation in healthy subjects was associated with less increase of upset feelings and better PASAT performance (Plewnia et al. 2015). Additionally, the authors found a negative correlation of increased upset feelings with PASAT performance. Further insight into underlying mechanisms of the PASAT comes from a recent study from our workgroup. We examined time dynamic neural signatures during PASAT performance by the means of feedback-locked event-related potentials (ERPs) in healthy subjects. We found valence-specific and stage-dependent associations of PASAT performance with the feedback-related negativity (FRN), P300, and the late positive potential (LPP), that provide the basis of further research into the mechanisms underlying the PASAT in depressed patients (Sommer et al. 2021).

The FRN is an early ERP peaking between 200 and 300 ms after feedback presentation at medial–frontal sites. It is sensitive to feedback valence and commonly found to be larger for negative than positive feedback (Ridderinkhof et al. 2004; Hajcak et al. 2006). Moreover, the FRN is assumed to reflect emotional reactions to negative feedback (Luu et al. 2003). Findings about the FRN in depressed patients are unequivocal. Some studies have found increased FRN amplitudes (Tucker et al. 2003; Santesso et al. 2008; Cavanagh et al. 2011), probably reflecting increased sensitivity and attention toward negative information, which matches a negativity bias in depressed patients. However, there are also findings pointing toward a reduced FRN amplitude in depressed patients (Foti and Hajcak 2009; Liu et al. 2014; Keren et al. 2018). It was suggested that this ambiguity could be explained by between patient variations regarding symptom severity and especially symptoms of anhedonia (Liu et al. 2014; Mueller et al. 2015). Indeed, Mueller et al. found that anhedonia in depressed patients attenuated neural reactions to negative feedback (Mueller et al. 2015). Moreover, this typical negative deflection after feedback is also referred to as reward positivity (RewP), i.e., a negative deflection that is more positive for rewards than losses (Proudfit 2015). In line with findings on reduced FRN amplitudes in depressed patients, depressive symptoms have been found to be linked to reduced RewP amplitudes (Proudfit, 2015; Weiß et al. 2020), reflecting reduced sensitivity to reward and positive feedback in depressed individuals (Proudfit et al. 2015). In healthy participants, we could replicate the typical finding of a larger amplitude after negative than positive feedback. Moreover, we found the valence-specific FRN (ΔFRN = negative − positive feedback) linked to task performance: larger neural activation after negative than positive feedback in the PASAT was associated with performance deteriorations pointing toward distraction by negative feedback in early feedback processing stages (Sommer et al. 2021).

The P300 is an ERP having its peak between 300 and 400 ms after stimulus presentation at centro-parietal sites, and is associated with attention allocation to task relevant and salient information, which includes content of emotional valence (Sutton et al. 1965; Polich, 2012). Moreover, it is assumed to reflect emotional involvement of the subject (Diner et al. 1985). In depressed patients commonly, a reduced P300 amplitude is found, which is associated with cognitive deficits as well as emotional and motivational abnormalities (Proudfit et al. 2015). This fits with our findings on the association of P300 amplitudes and PASAT performance in healthy subjects. Besides a larger amplitude after negative than positive feedback, we found reduced P300 activation after negative feedback to be associated with performance deteriorations. This probably reflects diminished resource allocation in later processing stages (Sommer et al. 2021).

The LPP is a positive deflection starting at about 200–300 ms after stimulus presentation that can persist for several seconds and is recorded at centro-parietal electrode sites (Cacioppo et al. 1996; Ito et al. 1998). The LPP is sensitive for emotional information, and enhanced for stimuli of positive and negative valence (Cuthbert et al. 2000). Moreover, it has been found to be regulated by CC mechanisms (Moser et al. 2006). The LPP and P300 have been shown to share features and in part reflect similar processes (Cuthbert et al. 2000). However, opposed to the P300, the LPP can outlast the stimulus presentation beyond several seconds. Thus, this sustained positive deflection captures continued processing of emotional content (Hajcak et al. 2009). Since CC deficits as well as emotional abnormalities are commonly found in MD, the LPP is an interesting ERP to study dysfunctional information processing in depressed patients and has been shown to be reduced for both, stimuli of positive and negative emotional valence (Blackburn et al. 1990; Foti et al. 2010; Proudfit et al. 2015; Klawohn et al. 2020). This indicates blunted emotional reactivity as well as motivational withdrawal and decreased cognitive engagement in depressed patients. In line with previous findings, showing larger LPP amplitudes after negative than positive feedback, we found an enhanced LPP for negative feedback compared to positive feedback during PASAT performance, probably reflecting the emotional impact of negative information. Moreover, although the association of the number of correct trials in the PASAT failed to reach significance, we found the performance stability, a measure of CC^1^ to be significantly correlated with the valence-specific LPP: a smaller LPP after negative than positive feedback was associated with reduced performance stability (Sommer et al. 2021). This probably reflects diminished resource allocation and motivational engagement. Our findings in healthy participants raise the question if similar relationships can be found in depressed patients, which could help to gain a better understanding of the mechanisms underlying the effectiveness of PASAT training for the reduction of depressive symptoms. Thus, the goal of our study is to use the neural signatures we found in healthy participants to investigate neural mechanisms of PASAT performance in depressed patients with the long-term aim to use these as measures of change in CC over the course of a CCT.

We hypothesized to find larger neural reactions to negative than positive feedback for all three feedback processing stages (FRN, P300, and LPP) reflecting increased attention allocation toward negative content in accordance with a negativity bias in depressed patients. Moreover, in view of our previous results and findings on ERPs in depressed patients, we assumed to find associations of depressive symptom severity, anhedonia, and PASAT performance with the valence-specific ERPs (ΔERP = positive − negative feedback). For the FRN, the assumptions about the direction of these associations were not clear. In line with our results in healthy subjects, larger ΔFRN probably would indicate increased distraction by negative feedback and associations with performance deteriorations and depressive symptom severity, consistent with increased sensitivity for negative feedback in depressed patients. However, as outlined above, former research has also found FRN and RewP to be reduced in depressed patients, suggesting a negative correlation of ΔFRN with PASAT performance, depressive symptom severity, and anhedonia. In line with findings of reduced P300 and LPP amplitudes in depressed patients that are associated with cognitive and motivational abnormalities, we hypothesize to find negative correlations of ΔLPP and ΔP300 with PASAT performance and positive correlations with depressive symptom severity and anhedonia. Moreover, since previous findings showed increased negative affect after PASAT performance and a correlation of this increase with performance deteriorations (Plewnia et al. 2015), we additionally hypothesize to replicate these findings in depressed patients and thus add evidence for the important role of CC of emotions for successful PASAT performance that seems to play a crucial role for the effectiveness of PASAT training for depression treatment.

## Materials and methods

Note that this study is part of a larger project (Clinical Trials Registration at clinicaltrials.gov, NCT03518749). Therefore, parts of the material and methods overlap with already published manuscripts (Sommer and Plewnia, 2021; Sommer et al. 2021). The data for the current paper were collected in the baseline session of a larger training study (Sommer and Plewnia, 2021). Thus, a detailed description of the materials and methods in part has been omitted in this manuscript.

### Tasks

The *PASAT* and the control task *color presentation (CP)* have been computer-based and implemented using Psycho-Py2 (v1.80.02; Peirce 2007, 2008).

In the PASAT, digits (1–9) were presented auditorily, initially with an interstimulus interval (ISI) of 3 s. Participants had to add the current digit to the digit they heard before, i.e., for each trial, exactly two numbers had to be added. Participants then indicated the result with the use of a keyboard equipped with the corresponding numbers (2–18). Simultaneously with the presentation of the next digit, subjects received feedback about the correctness of the previous trial indicated by green (correct) or red (incorrect) screen color. Difficulty of the task was adaptive as the ISI adjusted to the performance level of the subjects: after four consecutive correct (incorrect) trials, the ISI decreased (increased) by 100 ms. The task comprised three blocks, each lasting 5 min and breaks of 1 min between the blocks. Before the first block, all patients underwent 11 practice trials, which were excluded from analysis. The number of correct trails (*PASAT*_*corr*_) and the performance stability (*PASAT*_*PS*_) were used as the dependent variables. For the EEG analysis, only trials with a response were used (e.g., incorrect trials without a response were discarded).

Since the goal of the current study was to investigate neural reactions to feedback and not color information (which the feedback also contains), we additionally performed the control task *color presentation (CP)* (see Sommer et al. 2021). Participants were asked to sit in front of a monitor (distance: approximately 65 cm) and perceive red and green light peripheral by keeping their gaze on the keyboard just like they would do while performing the PASAT. The task consisted of two blocks each with a duration of 2.5 min. Red and green light was presented for 433 ms (as in the PASAT) in random order with a jittered interstimulus interval (1500–2500 ms).

### Electroencephalography

#### EEG procedure

For the electroencephalography (EEG) recording, an elastic cap (EASYCAP GmbH, Hersching, Germany) was equipped with active Ag/AgCl electrodes. EEG was registered from 27 scalp sites (FP1, F7, F3, Fz, F4, F8, FC5, FC1, FCz, FC2, FC6, C3, Cz, C4, CP5, CP1, CPz, CP2, CP6, P7, P3, Pz, P4, P8, O1, Oz, O2) using the actiCHamp amplifier system and the corresponding Brain Vision Recorder system (Brain Products GmbH, Gilching, Germany). To control for eye movements, an electrooculogram was recorded using electrodes placed on the lateral side of each eye (horizontal eye movements) and electrodes positioned approximately 1 cm below/above (Fp1) the left eye (vertical eye movements). Electrodes placed on the forehead and the left mastoid served as the ground and online reference, respectively. The online sampling rate was 1000 Hz. Impedances were kept below 10kΩ before initiation of the recording.

#### EEG analysis

We used the EEGLAB toolbox (Delorme and Makeig 2004) running on MATLAB 9.2 R2017a (The MathWorks, Natick, MA, USA) and the EEGLAB toolbox ERPLAP (Lopez-Calderon and Luck 2014) to analyze the EEG data. The data were resampled offline to 250 Hz and re-referenced to an average of the left and right mastoids and filtered using band-pass filters with a low and high cutoff of 0.1 and 35 Hz, and a notch filter at 50 Hz. Independent component analysis was used to manually remove ocular artifacts. Epochs ranging from -100 to 1000, locked to the onset of feedback (PASAT) and color (CP), were extracted. The automated artifact detection implemented in ERPLAB was used for artifact correction in the epoched EEG. On average 3.82% of the red feedback trials, 1.76% of the green feedback trials (PASAT) and 3.17% of the red and 2.46% of the green color trials (CP) were rejected from analysis due to excessive noise. Participants with more than 20% rejected trials were excluded from analysis (n = 5). Moreover, for one participant, the FP1 channel was removed due to excessive noise in this channel. Overall, 907 red feedback trials, 7820 green feedback trials (PASAT), and 3402 red and 3494 green color trials (CP) were used for the construction of the ERPs, which was done by separately averaging trials in the four conditions. For the analysis of the neural signatures of the PASAT, difference waves were calculated: positive feedback = green feedback (PASAT)—green color (CP), negative feedback = red feedback (PASAT)—red color (CP). All further ERP results refer to these difference waves. Time windows and electrode sites are the same as in our previous study (Sommer et al. 2021): the FRN was defined as the mean amplitude at Fz within a time window of 200–300 ms following feedback (Gehring and Willoughby, 2002). The P300 was scored as the base-to-peak difference in voltage between the most negative peak between 200 and 300 ms post-feedback and the most positive peak 300–400 ms post-feedback (Fabiani et al. 1987; Polich 1991; Polich and Kok 1995) of the average of three centro-parietal sites (Cz, CPz, Pz; Sutton et al. 1965; Johnson 1993). The LPP was defined as the mean amplitude between 400 and 1000 ms after feedback presentation at an average of five centro-parietal electrode sites (Cz, CP1, CPz, CP2, Pz; Hajcak et al. 2009; Weinberg and Hajcak 2010).

#### Procedure

Overall, 51 patients with a current depressive episode were enrolled. Five participants had to be removed due to excessive noise in the data during EEG recording. The data of the remaining 46 patients (age *M* = 37.50, *SD* = 14.19, 26 female) were analyzed. Although the sample size was determined by the number of patients enrolled in our larger project, two power analyses were conducted for the main analyses to ensure in advance that the planned analyses were adequately powered. For the correlation analyses, a sample size of 39 was estimated, based on the following values: *r* = 0.436 (based on the average of our previous results, Sommer et al. 2021), *α* = 0.05; *β* = 0.20. For the paired *t* tests, *N* = 23 was estimated, based on the following values: *d* = 0.64 (based on the average of our previous results, Sommer et al. 2021), *α* = 0.05; *β* = 0.20. Accordingly, even taking into account an additional tolerance of 20%, it could be assumed that the existing sample size was appropriate. All participants gave their written informed consent. Demographic and clinical characteristics of the sample are depicted in Table 1. Study eligibility was ascertained in a separate diagnostic session (see Sommer and Plewnia 2021 for inclusion criteria). In the EEG session (baseline session of the training), severity of depressive symptoms was assessed using a questionnaire (Beck Depression Inventory, BDI-II) and a semi-structured interview (Montgomery Asberg Depression Rating Scale, MADRS; Montgomery and Asberg 1979). Afterward, participants completed the PASAT and CP. Immediately before and after the PASAT, affect was assessed using the 20-item positive and negative affect schedule (PANAS; Krohne et al. 1996).

**Table 1:** Demographic and clinical characteristics of the sample

Characteristics	*N*	*M*	SD	Range
Sex (female/male)	26/20			
Age (in years)		37.50	14.19	19–62
Level of education: university entrance diploma (yes/no)	39/7			
Current psychotherapeutic intervention (yes/no)	27/19			
Duration of MDE in months		12.61	10.52	1–35
MADRS		27.98	6.42	15–45
BDI-II		25.91	7.64	12–47
PASAT_corr_		175.43	40.84	77–285
PASAT_PS_		58.69%	8.25%	32.53–71.72%
Antidepressive medication (yes/no)	27/19			

*PASAT*_*corr*_ number of correct trials in the PASAT, *PASAT*_*PS*_ performance stability, percent of consecutive correct responses relative to the overall correct responses

#### Statistical analysis

The statistical analyses were performed using SPSS (version 24.0). For all analyses, a 0.05 level of significance was employed. All statistical analyses were based on our previous study (Sommer et al. 2021) and chosen a priory based on our hypotheses. We used paired t tests to analyze changes in affect rating (PANAS before vs. after the PASAT) and to examine differences in neural activation after positive vs. negative feedback, separately for each ERP (FRN, P300, and LPP). Relationships between variables were analyzed using bivariate correlation analyses (Pearson correlation coefficient). This was done for the associations of the valence-specific neural activation (ΔERP = positive − negative feedback) with *PASAT*_*corr*_ and *PASAT*_*PS*_, changes in the affect ratings with *PASAT*_*corr*_ and *PASAT*_*PS*_ and severity of depressive symptoms (MADRS and BDI-II) and ΔERPs. Moreover, since previous research shows associations of anhedonia and ERP magnitudes, we additionally examined correlations of ΔERP and levels of anhedonia using bivariate correlation analyses (Pearson correlation coefficient). Anhedonia was assessed by means of item 8 of the MADRS (Inability to feel). Furthermore, as 58.7% of our participants received antidepressive medication that might influence neural activity, we additionally performed sensitivity analyses only including patients without psychotropic medication comprising all described ERP analyses (see above: *t* tests and correlational analyses) and comparisons of patients characteristics using independent *t* tests and Mann–Whitney *U* tests.

## Results

### Changes in affect rating

Affect significantly deteriorated after the PASAT as indicated by increased negative affect (before: *M* = 15.02, *SD* = 4.33, after: *M* = 23.41, *SD* = 9.24; *t*_(45)_ = 7.354, *p* < 0.001). There was no significant change in positive affect (before: *M* = 21.83, *SD* = 5.69, after: *M* = 23.20, *SD* = 6.73; *t*_(45)_ = 1.487, *p* = 0.144]. Moreover, there were no significant correlations of the affect ratings with *PASAT*_*corr*_ and *PASAT*_*PS*_ (all *p* ≥ . 088).

### Electrophysiological data

#### Feedback-related negativity

**Fig. 1:**
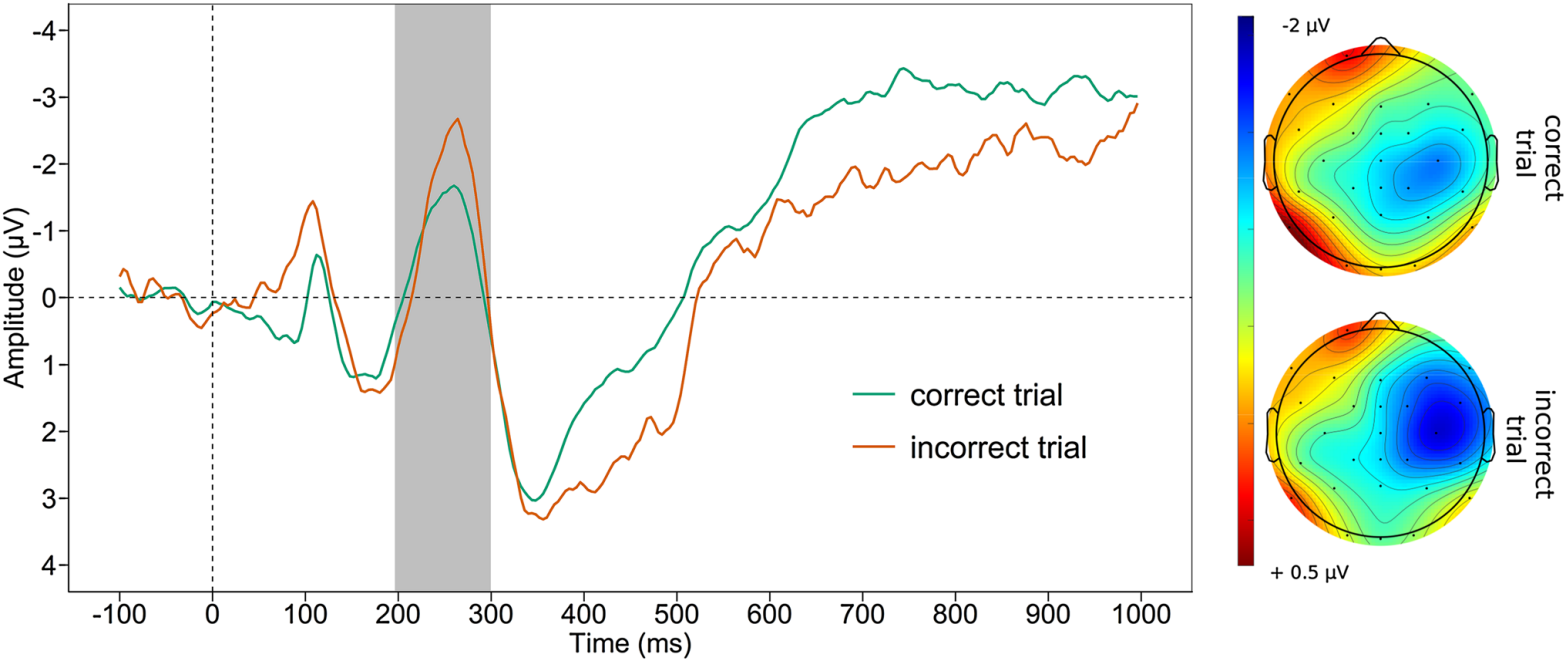
Feedback-related negativity*.* Grand average waveform at Fz and scalp map displaying the mean voltage distribution (200–300 ms post-feedback) of the feedback-related negativity, separately for negative and positive feedbacks

See Fig. 1 for the grand average waveform of the FRN and the mean voltage distribution across the scalp, separately for negative and positive feedback. There was no significant difference between neural activation after positive (*M* =  − 0.846, *SD* = 2.592) and negative feedback (*M* =  − 1.226, *SD* = 3.103, *t*_(45)_ = 1.047, *p* = 0.300). Additionally, there was no significant associations of ΔFRN with the *PASAT*_*corr*_ and *PASAT*_*PS*_ nor depression scores or symptoms of anhedonia (all *p* ≥ 0.179, see Table 2).

**Table 2:** Correlations of ΔERPs with PASAT scores and depressive symptoms

	PASAT_corr_	PASAT_PS_	MADRS	BDI-II	Anhedonia
ΔFRN	*r* = .105, *p* = .486	*r* = .202, *p* = .179	*r* = − .110, *p* = .467	*r* = − .027, *p* = .860	*r* = − .194, *p* = .196
ΔP300	*r* = .051, *p* = .739	*r* = .035, *p* = .816	*r* = − .200, *p* = .182	*r* = − .249, *p* = .095	*r* = − .171, *p* = .255
ΔLPP	*r* = − .185, *p* = .219	*r* = .026, *p* = .864	*r* = − .194, *p* = .195	*r* = − .033, *p* = .828	*r* = − .216, *p* = .149

*PASAT*_*corr*_ number of correct trials in the PASAT, *PASAT*_*PS*_ performance stability, percent of consecutive correct responses relative to the overall correct responses

#### P300

**Fig. 2:**
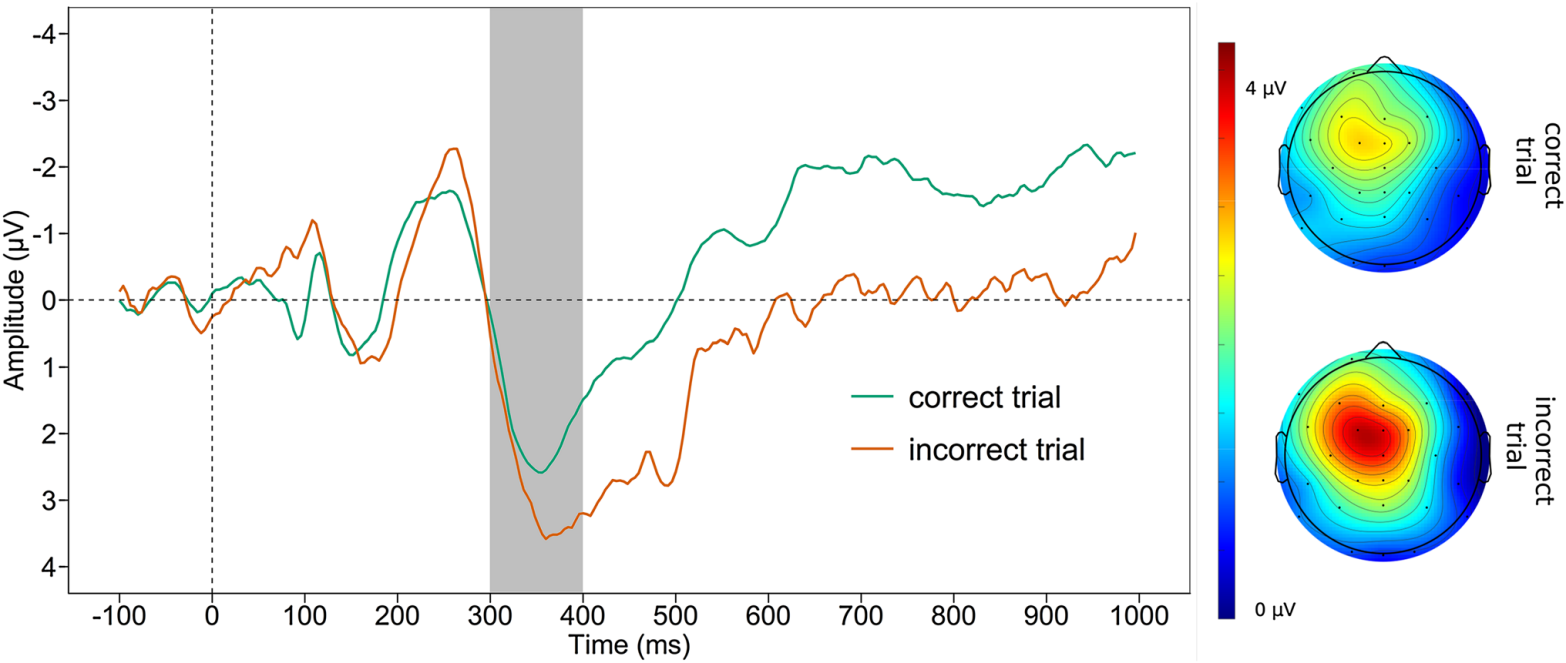
P300. Grand average waveform averaged across Cz, CPz, Pz, and scalp map displaying the mean voltage distribution (300–400 ms post-feedback) separately for negative and positive feedbacks of the P300

Figure 2 displays the grand average waveform of the P300 and the mean voltage distribution across the scalp separately for negative and positive feedback. For the P300, neural activation after negative feedback (*M* = 9.982, *SD* = 3.769) was larger than after positive feedback (*M* = 6.282, *SD* = 2.337, *t*_(45)_ = 8.976, *p* < 0.001). There were no significant correlations of ΔP300 with *PASAT*_*corr*_ and *PASAT*_*PS*_, nor depression scores or symptoms of anhedonia (all *p* ≥ 0.095, see Table 2).

#### Late positive potential

**Fig. 3:**
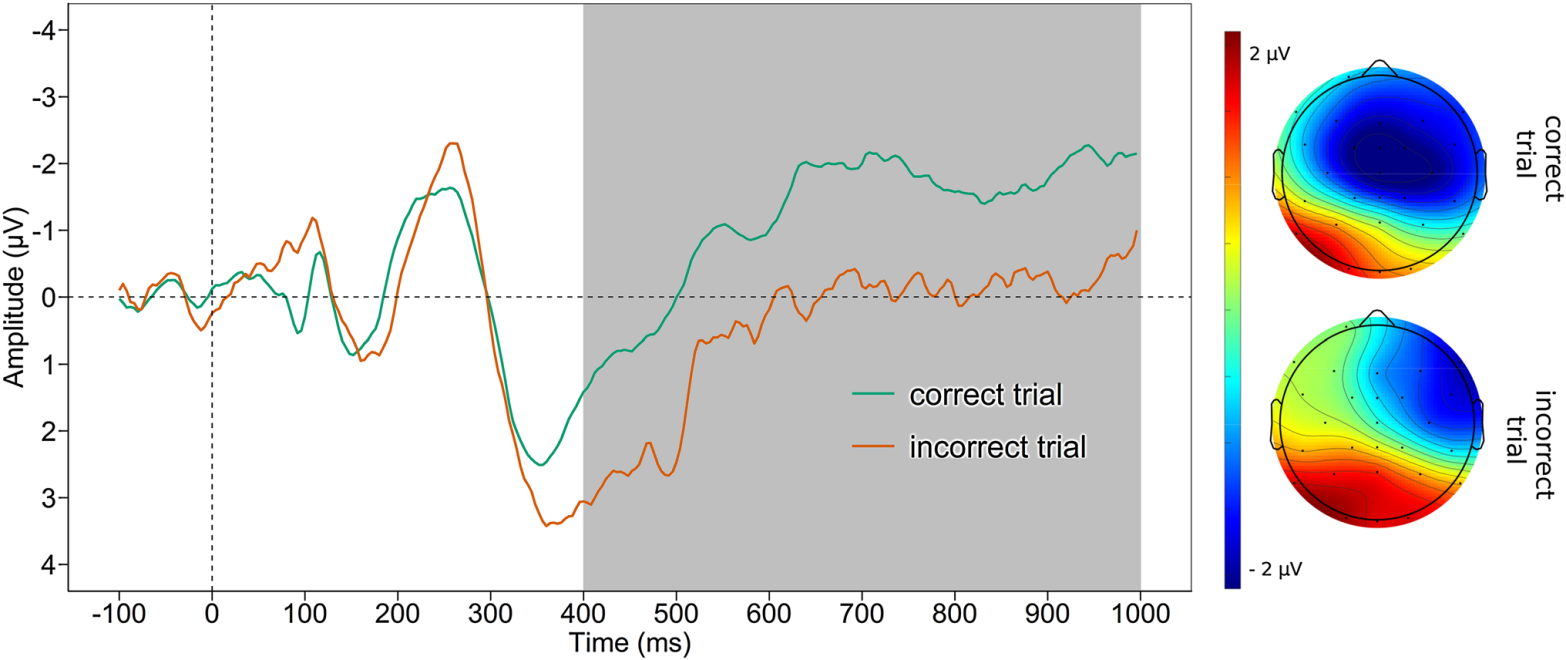
Late positive potential*.* Grand average waveform averaged across Cz, CPz, Pz, CP1, CP2, and scalp map displaying the mean voltage distribution (300–400 ms post-feedback) separately for negative and positive feedbacks of the LPP

See Fig. 3 for the grand average waveform of the LPP and the mean voltage distribution across the scalp separately for negative and positive feedback. For the LPP, a larger activation after negative (*M* = 0.443, *SD* = 4.440) than positive feedback (*M* =  − 1.220, *SD* = 2.579) was found (*t*_(45)_ = 3.573, *p* = 0.001). There were no significant association of the ΔLPP with *PASAT*_*corr*_ and *PASAT*_*PS*_ nor depression scores or symptoms of anhedonia (all *p* ≥ 0.195, see Table 2).

#### Effects of medication

The following differences in results compared to the complete sample were observed: the FRN in unmedicated patients was marginal significant larger after negative feedback (*M* =  − 2.245, *SD* = 3.376) than after positive feedback (*M* =  − 1.145, *SD* = 2.126, *t*_(18)_ = 1.987, *p* = 0.062). Additionally, there was no longer a significant difference in amplitudes between negative (*M* =  − 0.566, *SD* = 5.321) and positive feedback (*M* =  − 1.475, *SD* = 2.704, *t*_(18)_ = 0.953, *p* = 0.353) for the LPP. Moreover, in unmedicated patients, a significant correlation of ΔFRN with *PASAT*_*PS*_ was observed (*r* = 0.533, *p* = 0.019). A smaller ΔFRN was associated with a more stable performance in the PASAT. For the ΔP300, we found a significant correlation with the BDI-II and anhedonia in unmedicated patients. Higher BDI-II and anhedonia scores were associated with smaller ΔP300 (*r* =  − 0.480, *p* = 0.038 and *r* =  − 0.517, *p* = 0.023, respectively).

Additionally, unmedicated patients showed significantly more correct trials in the PASAT (unmedicated: *M* = 189.42, *SD* = 34.80, medicated: *M* = 165.59, *SD* = 42.49, *t*_(44)_ = 2.013, *p* = 0.05) and a more stable performance (unmedicated: *M* = 62.20%, *SD* = 6.00, medicated: *M* = 56.20%, *SD* = 8.80, *t*_(44)_ = , 2.575 *p* = 0.013). All other outcomes did not differ between medicated and unmedicated patients. However, several differences between unmedicated and medicated patients concerning patient characteristics are observable (see Table 3).

**Table 3:** Demographic and clinical characteristics of the sample for medicated and unmedicated patients separately

Characteristics	*N*	*M* (SD)	*N*	*M* (SD)	Test statistic
	Unmedicated patients (*N* = 19)		Medicated patients (*N* = 27)		
Sex (female/male)	14/5		15/12		*χ*^2^ = 3.80, *p* = .051
Age (in years)		34.42 (14.92)		39.67 (13.51)	*t*_(44)_ = 1.242, *p* = .221
Level of education: university entrance diploma (yes/no)	19 /0		20/7		*χ*^2^ = 5.68, *p* = .017*
Current psychotherapeutic intervention (yes/no)	7/12		20/7		*χ*^2^ = 6.24, *p* = .013*
Duration of MDE in months		16.53 (12.39)		9.85 (8.15)	*t*_(44)_ = 2.056, *p* = .049*
MADRS		26.89 (6.50)		28.74 (6.38)	*t*_(44)_ = 0.959, *p* = .343
BDI-II		26.21 (6.89)		25.70 (8.24)	*t*_(44)_ = 0.219, *p* = .827
PASAT_corr_		189.42 (34.80)		165.59 (42.49)	*t*_(44)_ = 2.013, *p* = .05*
PASAT_PS_		62.20% (6.00)		56.2% (8.80)	*t*_(44)_ = , 2.575 *p* = .013*
Anhedonia		2.63 (1.46)		3.07 (1.52)	*t*_(44)_ = 0.989, *p* = .328

*PASAT*_*corr*_ number of correct trials in the PASAT, *PASAT*_*PS*_ performance stability, percent of consecutive correct responses relative to the overall correct responses

*Difference is significant

## Discussion

The goal of the current study was to investigate neural mechanisms underlying CCTs for depressive symptoms by means of neural signatures of the PASAT found in healthy participants. In line with our hypotheses, we found negative affect significantly increased after PASAT performance; this change, however, was not correlated with PASAT performance. As expected, neural activation after negative feedback was larger than after positive feedback for the P300 and LPP. This difference was not significant for the FRN. Furthermore, opposed to our hypotheses, we could not find any associations of the valence-specific ERPs (ΔFRN, ΔP300, ΔLPP) with PASAT performance, depression scores, or symptoms of anhedonia.

In contrast to our findings in healthy subjects, the FRN after negative feedback was not larger than after positive feedback. Such reduced ΔFRN amplitudes are in accordance with the previous findings about diminished FRN amplitudes in depressed patients (Foti and Hajcak 2009; Liu et al. 2014; Keren et al. 2018), and might reflect emotional indifference and motivational withdrawal as suggested by Mueller et al. (Mueller et al. 2015). However, in relation to our previous findings in healthy subjects, this is quite unexpected, since we have found increased FRN amplitudes to be associated with performance deteriorations, indicating diminished top–down-driven CC over distraction by negative feedback. Thus, it could have been assumed that reduced CC in depressed patients goes along with increased FRN amplitudes reflecting attentional engagement with negative information which distracts from goal-oriented behavior, e.g., PASAT performance. However, our results do not support this hypothesis. Rather, the lack of correlation between PASAT performance and FRN amplitudes suggests that the relationships of neural signatures with CC found in healthy subjects are not applicable to our depressed sample, probably due to pathophysiological characteristics. Moreover, the previous studies have shown links of increased depression severity and symptoms of anhedonia with reduced FRN amplitudes (Mueller et al. 2015). In our study, we could not replicate this finding. In this context, it has to be noted that the PASAT differs from usual tasks used to elicit FRN amplitudes in the way that feedback is presented simultaneously with the next target. Thus, the processing of the next digit might have influenced FRN amplitudes and obscured associations with depressive symptoms. Additionally, differences in measures of anhedonia might account for the missing correlation of anhedonia with the FRN. Whereas we used item number 8 of the MADRS interview, Mueller et al. used the Anhedonic Depression subscale from the Mood and Anxiety Symptom Questionnaire, which might be a more sensitive measure for anhedonia.

For the P300 and LPP, larger neural activation after negative than positive feedback was found. This is in line with our hypothesis, indicating increased attention allocation toward information of negative content. However, just like for the FRN, no association with PASAT performance was found for the P300 nor LPP. Thus, increased attention allocation after negative feedback seems to be unrelated to increased resource allocation. Depressed patients seem to be unable to use the neural activation of late processing stages after negative feedback information to adapt accordingly. Moreover, the previous studies have found associations of reduced P300 (Gangadhar et al. 1993; Nan et al. 2018) and LPP (Blackburn et al. 1990; Foti et al. 2010; Proudfit et al. 2015; Klawohn et al. 2020) amplitudes with depressive symptoms. This is not in line with our results, since no significant correlative relationships of depression severity and P300 nor LPP amplitudes were found. A major difference to these studies is the use of performance feedback in our study as stimuli which might explain this difference. Previous studies have used words of emotional valence, pictures of pleasant or unpleasant content, or monetary gains or losses. Overall, this indicates that there is no relationship between symptoms of depression and neural signatures in later stages of performance feedback processing in the PASAT.

Differences of neural signatures during PASAT performance in depressed patients compared to healthy subjects (Sommer et al. 2021) may have several causes. A pathophysiological characteristic that may be reflected by our findings is a loss of specificity of neural activation to feedback parallel to the processing of new information (i.e., new target digit) which might be related to cognitive overload in depressed patients. Thus, patients may no longer be able to process feedback in an orderly manner that allows for the best possible use of feedback information. Moreover, due to the diagnostic system for psychiatric diseases based on symptoms, not brain-based biological alterations (Fischer 2012), there is a high between patient variability of neural activation and brain alterations that might obscure consistent pathological processes distinctive for certain types of MDD. Furthermore, besides specific pathophysiological characteristics in depressed patients, reasons for these differences for all processing stages (FRN, P300, and LPP) could be differences in tasks between our studies. To avoid ceiling effects in healthy participants, we used a more challenging 2-back version of the PASAT to derive ERPs during PASAT performance in our previous study. In the 2-back PASAT, participants have to add the digit before the last one to the currently presented one. Therefore, cognitive functions involved differ to some extent between the tasks, which might be reflected in differences in ERPs. In contrast to the one-back PASAT, the 2-back PASAT puts higher demands on working memory, thus increasing task difficulty. Moreover, this could also influence expectancy of negative feedback. Supposedly, in the 2-back PASAT, participants often are not sure if the remembered digit is the correct one, whereas in the one-back PASAT, mistakes are more often due to speeded response. It can be assumed that in the latter, participants already expect to receive negative feedback which is not the case if one is unsure if the remembered digit is the correct one. This is especially relevant for the FRN as it has been shown that its amplitude is significantly larger for unexpected than expected negative feedback (Bellebaum and Daum 2008; Weismüller and Bellebaum 2016).

Furthermore, our results suggest that psychotropic medication influences neural activation. Interestingly, for unmedicated patients, neural signatures in early feedback processing stages (FRN) are more similar to healthy subjects, as the FRN shows a tendency for a larger neural activation after negative than positive feedback and was linked to a more stable performance (as in Sommer et al. 2021). However, for the LPP, no longer a differential neural reaction to negative and positive feedback was found, suggesting a blunted emotional responses in unmedicated patients for very late processing stages. In line with our hypotheses, increased depressive symptoms (BDI-II) and anhedonia were associated with smaller ΔP300, indicating that in unmedicated patients, more severe depressive symptoms and the inability to feel are associated with indifference toward negative feedback, since neural signatures for negative and positive feedback become similar. Overall, these results support the assumption that psychotropic medication influences neural activation associated with cognitive control and the processing of emotionally valenced content, and suggest that future studies should take that into account. However, based on this additional sensitivity analysis, we cannot conclude that the differences are actually caused by the medication. Thus, the examination of the specific drug effects on CC-related neural activation should be subject of future studies.

As expected, negative affect significantly increased after the PASAT. This is in line with findings from Plewnia and colleagues (Plewnia et al. 2015) about the impact of PASAT performance on mood in healthy subjects. In addition, the authors observed a link between elevated scores of upset feelings and performance deteriorations, which was interpreted as diminished CC over negative emotions. In our previous trial (Sommer et al. 2021) and in the current study involving subjects with depression, we were not able to replicate this finding. This might indicate that the PANAS is not sensitive enough to detect latent affect changes. Moreover, in depressed patients, PASAT performance might be more significantly influenced by other variables, such as lack of motivation and working memory dysfunctions, than CC over emotions. This should be investigated in future studies to gain further information on the effectiveness of PASAT training.

The small number of negative feedback trials used to build the ERPs depicts a limitation of our study. On average, only about 20 negative feedback trials per subject were included in the analysis, possibly resulting in increased noise due to artifact-heavy trials. However, due to the highly demanding nature of the task, an increasement of trials overall was not possible without overstraining our sensitive sample.

Taken together, due to the missing associations of ERPs, CC and depressive symptoms only limited understanding of the mechanisms underlying the effectiveness of PASAT training for the reduction of depressive symptoms can be gained. Accordingly, our results do not support the idea to use ERPs during PASAT performance as measures of change in CC over the course of a CCT. Nevertheless, despite the limited explanatory power of our results, this study makes an important contribution to the field by exploring hypotheses and open research questions based on previous findings. Moreover, our results indicate significant influences of psychotropic medication on ERPs and thus inform future studies investigating neural signatures in psychiatric samples.

## Data Availability

Data and scripts underlying our findings are available on https://osf.io/xmz7h/.

## Footnotes

1 Performance stability: percent of consecutive correct responses relative to the overall correct responses (see (Sommer et al. 2021).
